# Modulation of NMDA-Mediated Clock Resetting in the Suprachiasmatic Nuclei of *mPer2*^Luc^ Mouse by Endocannabinoids

**DOI:** 10.3389/fphys.2019.00361

**Published:** 2019-03-29

**Authors:** Martin Sládek, Alena Sumová

**Affiliations:** Institute of Physiology, Academy of Sciences of the Czech Republic, Prague, Czechia

**Keywords:** circadian, suprachiasmatic nucleus, entrainment, NMDA, glutamate receptor, endocannabinoids, PER2::LUC, phase response curve

## Abstract

Light entrains the master circadian clock in the suprachiasmatic nucleus (SCN) predominantly through glutamatergic signaling via NMDA receptors. The magnitude and the direction of resulting phase shifts depend on timing of the photic stimulus. Previous reports based on behavioral and electrophysiological data suggested that endocannabinoids (EC) might reduce the ability of the SCN clock to respond to light. However, there is little direct evidence for the involvement of EC in entrainment of the rhythmic clock gene expression in the SCN. We have used luminescence recording of cultured SCN slices from *mPer2*^Luc^ mice to construct a complete phase response curve (PRC) for NMDA receptor activation. The results demonstrated that NMDA administration phase-shifts the PER2 rhythm in a time-specific manner. A stable “singularity,” in the course of which the clock seemingly stops while the overall phase is caught between delays and advances, can occur in response to NMDA at a narrow interval during the PER2 level decrease. NMDA-induced phase delays were affected neither by the agonist (WIN 55,212-2 mesylate) nor by the antagonist (rimonabant hydrochloride) of EC receptors. However, the agonist significantly reduced the NMDA-induced phase advance of the clock, while the antagonist enhanced the phase advance, causing a shift in the sensitivity window of the SCN to NMDA. The modulation of EC signaling in the SCN had no effect by itself on the phase of the PER2 rhythm. The results provide evidence for a modulatory role of EC in photic entrainment of the circadian clock in the SCN.

## Introduction

A principal pacemaker in the suprachiasmatic nucleus (SCN) of the hypothalamus governs the mammalian circadian system (Ralph et al., 1990). While transcriptional/translational feedback loops of families of clock genes (Mohawk and Takahashi, 2011), such as *Per*, *Cry*, *Clock*, *Bmal*, *Rev-Erb*, and *Ror*, generate oscillations in individual neurons (Welsh and Reppert, 1996) and astrocytes (Brancaccio et al., 2017), the complex network within the SCN is responsible for robustness and plasticity of the overall circadian rhythm (Hastings et al., 2014).

The neuronal subpopulations in the SCN vary in their main neurotransmitters, represented mainly by γ-aminobutyric acid (GABA, entire SCN), vasopressin (AVP, dorsomedial shell region, dmSCN) and vasoactive intestinal polypeptide (VIP, ventrolateral core region, vlSCN) (Welsh et al., 2010). The retinohypothalamic tract (RHT) connects retina with the vlSCN, conveys light information from the external environment and entrains the SCN to the actual light-dark cycle (Moore and Lenn, 1972). Glutamate is the main neurotransmitter released from RHT upon light exposure (Van den Pol, 1991). It binds to ionotropic glutamatergic receptors (iGluR) present in the SCN, that is mainly the N-methyl D-aspartate receptors (NMDAR) (Ding et al., 1994), but also to the α-amino-3-hydroxy-5-methylisoxazole-4-propionic acid receptors (AMPAR) (Gannon and Rea, 1994). Their activation induces a cascade of transient and sustained responses that entrain the circadian pacemaker (Ding et al., 1998; Meijer and Schwartz, 2003; Albers et al., 2017). SCN clock reacts to a photic stimulus mainly by adjusting its phase due to cAMP response element-binding protein phosphorylation (Gau et al., 2002) and subsequent changes in clock gene expression (Challet et al., 2003). The phase adjustment has previously been described after exposure to light *in vivo* (Daan and Pittendrigh, 1976), as well as by *in vitro* activation of both the NMDAR (Colwell and Menaker, 1992; Shibata et al., 1994) and the AMPAR (Mizoro et al., 2010). Importantly, light induces phase delays in the early subjective night, phase advances in the late subjective night, but only small phase shifts during the subjective day, in part due to time-dependent changes in NMDAR activity (Pennartz et al., 2001). The phase shifts can be plotted as a function of circadian phase of the stimulus to construct phase response curve (PRC) (Johnson, 1992).

Endocannabinoids (EC) such as anandamide (AEA) and 2-arachidonoylglycerol (2-AG), endogenous analogs of psychoactive phytocannabinoids present in marijuana (Lu and Mackie, 2016), belong among the substantial number of hypothalamic neurotransmitters. EC bind to cannabinoid receptor 1 (CB1R), that is robustly expressed in various hypothalamic nuclei (Wittmann et al., 2007), including a subset of vlSCN neurons (Sanford et al., 2008; Acuna-Goycolea et al., 2010). Interestingly, previous studies have shown that signaling via CB1R inhibits light-induced phase shifts of wheel-running behavior in hamsters (Sanford et al., 2008) and mice (Acuna-Goycolea et al., 2010). However, the effects of EC on the circadian phase of the SCN clock and their effects of the photic entrainment pathways impinging on the clock gene expression are unknown.

To examine the role of EC signaling in the modulation of the SCN phase response to light, we used *in vitro* recordings of circadian luminescence of organotypic SCN explants from *mPer2*^Luc^ knockin mice (Yoo et al., 2004). The paper presents detailed PRCs to NMDAR activation and shows that local stimulation or inhibition of EC signaling have opposing modulatory effects on the phase response to light-mimicking stimulus depending on the immediate phase of the SCN clock.

## Materials and Methods

### Animals

Adult male *mPer2*^Luc^ mice (strain B6.129S6-Per2tm1Jt/J, IMSR_JAX:006852, The Jackson Laboratory, Bar Harbor, United States) aged 3–12 months were used for the experiments (females were used only rarely; no significant differences between both sexes were detected). The mice were housed individually under a light/dark cycle with 12 h of light and 12 h of darkness (LD12:12; the lights were on between 07:00 and 19:00) in a temperature-controlled facility at 23 ± 2°C with free access to food and water. All experiments were approved by the Animal Care and Use Committee of the Institute of Physiology and were in agreement with the Animal Protection Law of the Czech Republic, as well as the European Community Council directive 2010/63/EU. All efforts were made to ameliorate the suffering of the animals.

### Preparation of Organotypic Explants and Luminescence Recording

The mice were sacrificed between 12:00 and 15:00 via rapid cervical dislocation. Hypothalamic regions containing the SCN were sliced on vibratome (VT1200S, Leica, Wetzlar, Germany, two 250 μm slices/mice). The explants were immediately placed onto Millicell Culture Inserts (Merck, Darmstadt, Germany) inside 35 mm Petri dishes with 1 ml of air-buffered recording medium (Yamazaki and Takahashi, 2005) supplemented with 100 U/ml penicillin, 100 μg/ml streptomycin, 1x GlutaMAX (Thermo Fisher Scientific, Waltham, MA, United States), 5% fetal calf serum (Merck), 1% B27 supplement (ThermoFisher) and 0.1 mM D-Luciferin (Biosynth, Staad, Switzerland). Explant cultures were allowed to settle *in vitro* for at least 14 days before recording started in Lumicycle (Actimetrics, United States). After 3 days of recording, samples were either treated with 30 μM N-methyl-D-aspartate (NMDA, glutamate receptor agonist, diluted in culture medium, Merck) or vehicle (same volume of culture medium), or pretreated 30 min in advance with 10 μM WIN 55,212-2 mesylate (WIN, cannabinoid receptor agonist, in DMSO, Merck) or 10 μM rimonabant hydrochloride/SR 141716A (RIM, cannabinoid receptor antagonist, in DMSO, Merck) and then treated with NMDA. To test the effects of WIN and RIM alone, drugs were applied directly to the medium and DMSO (0.04% final concentration in the medium) was used as a vehicle. To minimize unavoidable temperature changes, inserts were only briefly (<20 s) removed from the medium and kept on a warm (37°C) dish while a small volume of drug was applied to the medium. Due to small differences in endogenous periods *in vitro*, individual explants in a single experiment differed in their phase at the time of the treatment. To analyze the phase response during the entire circadian cycle, we repeated the experiments multiple times at different time points with different explants. To increase n, while keeping the amount of sacrificed animals as low as possible, selected explants were washed in warmed (37°C) PBS (Merck) twice for 5 min and kept undisturbed in recording medium for 3–4 weeks before repeating the treatment. Experiment 1 (NMDA, VEH) included 70 previously untreated and 41 reused explants from 65 individual mice, experiment 2A-D (WIN, RIM, VEH) included 31 previously untreated and 135 reused explants from 90 mice, while experiment 2E (NMDA + WIN, NMDA + RIM) included 59 previously untreated and 34 reused explants from 59 mice.

### Data Analysis and Statistics

The phase shifts were quantified by fitting a sine curve to the first three full circadian cycles of a 24 h running average baseline-subtracted rhythm and then extrapolating beyond the time of the treatment. The resulting phase shift was calculated as a difference between the extrapolated sine curve (reflecting the original phase) and the actual measured phase after the treatment, normalized to endogenous period after the stimulus, and designated as either a phase advance (+) or a phase delay (−). The PRC was constructed by plotting the calculated relative phase shift as a function of the time of the treatment normalized to the endogenous period of the SCN before the treatment and expressed relative to the trough (time 0) and peak (time 12) of the rhythm. For statistical comparisons of continuous PRCs, data were binned in 3 h intervals. The phase transition curve (PTC) was constructed by plotting the peak of the first full cycle after the treatment (y, new phase) as a function of the corresponding peak of the extrapolated sine curve (x, old phase). The PTC data were tetra-plotted for clarity. Amplitude and period were quantified by sine curve fitting three full cycles before and after stimulus and expressed X_AFTER_/X_BEFORE_. Only samples with relative amplitude >4 and high integrity of the oscillations were analyzed; two explants that showed close to zero amplitude after NMDA (discussed in section NMDAR Activation During a Narrow Window of SCN Sensitivity Can Disrupt Overall PER2 Rhythm) were excluded from the analysis, because their phase shifts were impossible to calculate. Continuous PRC was analyzed by two-way ANOVA with Šidák’s multiple comparisons, while relative amplitude was analyzed by cosinor as detailed previously (Polidarova et al., 2014). Circadian luminescence levels, period and phase change in Figure 2A–C were calculated by analyzing the parameters 3 cycles before and 3 cycles after a drug application and expressed as X_AFTER_/X_BEFORE_; the data were analyzed by one-way ANOVA with Dunnett’s multiple comparisons test. *P* < 0.05 was required for reporting significance. All statistical tests were performed in Prism 7 (GraphPad, United States).

## Results and Discussion

### NMDAR Activation During a Narrow Window of SCN Sensitivity Can Disrupt Overall PER2 Rhythm

**FIGURE 1:**
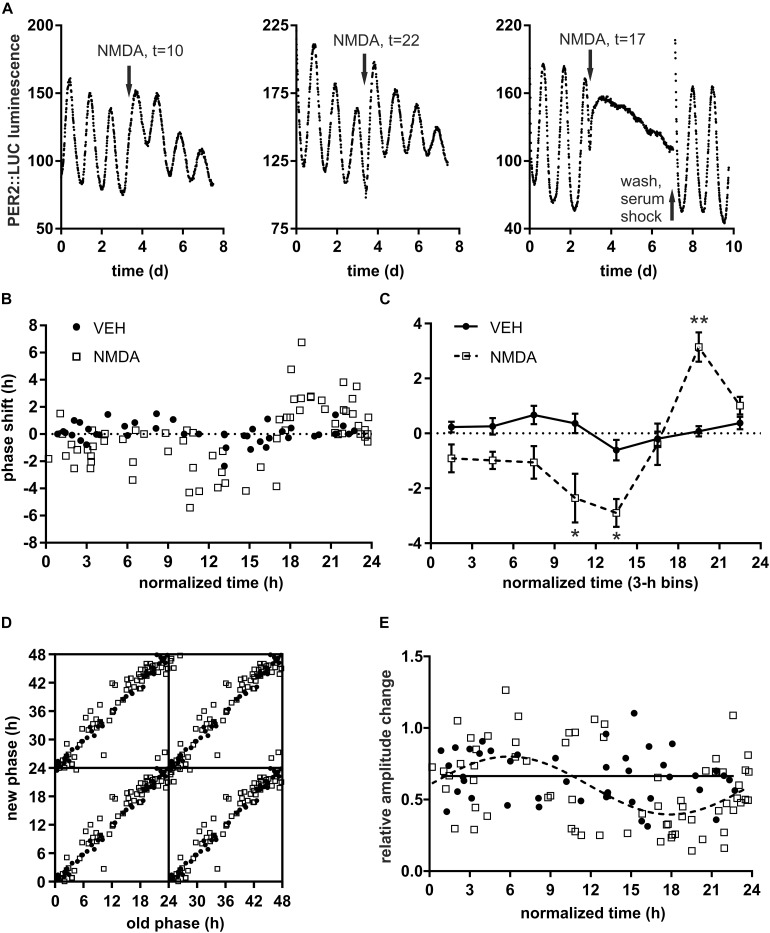
Quantitative analysis SCN phase response to NMDA (N-methyl D-aspartate) receptor activation. **(A)** Representative PER2 luminescence traces after treatment of SCN explants by 30 μM NMDA at different normalized time points (arrow). Normalized time of the treatment (*t*) is defined relative to previous trough of PER2 luminescence (i.e., *t* = 0 h at minimum and *t* = 12 h at peak of PER2 levels, 1 h = period of the explant before the treatment/24). Approximately 10% (*n* = 2 out of 19) explants treated between *t* = 16–20 h (i.e., around the inflection point between max and min of luminescence) lost detectable overt rhythms; NMDA wash out and 50% serum shock rescued the rhythmic coherence; these were not included in the phase shift quantification. **(B)** Phase response to NMDA (empty squares, *n* = 68) or VEH (full circles, *n* = 43). X-axis – normalized time of the treatment, Y-axis – relative phase shift (1 h is defined as the period of the explant after the treatment/24; positive values = advance, negative values = delay) of the PER2 luminescence. **(C)** Phase response curve (PRC) constructed from data in **B** by binning across 3 h intervals. Asterisks show results of Šidák’s multiple comparisons test between phase shifts in response to NMDA vs. VEH at corresponding time points. ^∗^*P* < 0.05, ^∗∗^*P* < 0.005; *n*_VEH_ = 3–8, *n*_NMDA_ = 6–17/time point. **(D)** Phase transition curve (PTC) of PER2 luminescence after NMDA or VEH treatment. Data expressed as “new phase” (measured during the first full cycle after the treatment) vs. “old phase” (i.e., extrapolated corresponding phase before the treatment) and tetraplotted for clarity. **(E)** Relative amplitude change (amplitude after/before the treatment with NMDA or VEH). Cosinor analysis (full line = VEH, dashed line = NMDA) showed significant (*P* = 0.0005) variation in amplitude after NMDA treatment at different time points.

In the first step, we examined the cultured mouse SCN phase responses to a light-mimicking stimulus, NMDAR activation, by exploiting PER2-driven luminescence rhythm. The phase shifts resulting from administration of NMDA and vehicle at various time points allowed construction of detailed PRCs. The circadian rhythm in PER2 expression shifted its phase after NMDAR stimulation (Figure 1A). Similar results were previously reported after stimulation of NMDAR in *Per1*^Luc^ mice (Asai et al., 2001) or AMPAR in *Per2*^Luc^ mice (Mizoro et al., 2010). NMDA stimulus phase-delayed the clock when applied 8–16 h after the start of the preceding luminescence cycle (t), phase-advanced the clock when applied at *t* = 16–24 and had little effect when applied at *t* = 0–8. Interestingly, NMDA treatment around *t* = 17 rendered 2 explants (out of 19 examined around that time point) almost completely arrhythmic (Figure 1A). We restored the overall rhythm in the arrhythmic slice by washing off the NMDA and subjecting it to 50% serum shock for 1.5 h (Figure 1A, right), demonstrating the intact viability of the SCN after the treatment. The lack of detectable rhythm was likely due to stable out-of-phase cycling of equal amount of SCN cells or their subpopulations rather than a loss of oscillations at the cellular level (Pulivarthy et al., 2007), although imaging evidence would be required to rule out the latter possibility. Thus, the NMDA applied *in vitro* is able to induce 6 h or even larger phase shifts in both directions simultaneously in a significant number of SCN cells when applied during a very narrow window of opportunity around the falling inflection point of PER2 luminescence.

This “singularity” or zero-amplitude behavior of the oscillator in response to light pulse around midpoint of subjective night was previously described in mammals and other model organisms by using locomotor activity monitoring (Winfree, 1970; Jewett et al., 1991; Honma and Honma, 1999) and luminescence recording of photosensitive cells (Ukai et al., 2007). It can also manifest for example in response to VIP signaling in the SCN (An et al., 2013) or in response to stress in the kidney (Tahara et al., 2017) of *Per2*^Luc^ mice. We cannot exclude the possibility that continuous presence of NMDA in the culture medium has unforeseen long-term effects on the SCN due to increased activity of NMDARs that might account for the lack of detectable rhythm. Nevertheless, explants treated with NMDA at different time points still showed high integrity rhythms over several days of measurement. Therefore, we believe that NMDA acted as a strong entraining stimulus that symmetrically dispersed the phases of individual cellular oscillators, affecting the overall amplitude of the SCN clock as discussed in the next chapter.

### Phase Response and Amplitude Response to NMDAR Activation

Expressing the magnitude and direction of phase shifts as a function of time of the treatment with NMDA or VEH (Figure 1B) and binning across 3 h intervals (Figure 1C) allowed us to visualize PRC of the SCN clock. The shape of the PRC closely resembled that of a typical type 1 (Johnson, 1992) phase response described previously for either light stimulus (VanderLeest et al., 2009) or NMDA microinjection (Mintz et al., 1999) and quantified with the use of locomotor activity monitoring. In our study the PRC to NMDA was significantly different when compared with the PRC to VEH (2-way ANOVA, *P*_interaction_ < 0.0001, *P*_drug_ = 0.0099, *F*(1, 95)_drug_ = 6.92). The curves did not differ during interval *t* = 0–8, suggesting this interval of PER2 expression corresponds to subjective day “dead zone” *in vivo* and marks the gating of the SCN response to NMDAR activation. Average phase delays were the largest between *t* = 9–15 [Šidák’s multiple comparisons test NMDA vs. VEH, *P*_9–12_ = 0.023, *t*(95)_9–12_ = 3.06, *P*_12–15_ = 0.022, *t*(95)_12–15_ = 3.07] and advances were the largest between *t* = 18–21 [*P*_18–21_ = 0.0045, *t*(95)_18–21_ = 3.57]. The mean magnitude of the NMDA-induced phase shift was around 3 h in both directions but reached up to 6 h in individual slices during the highest sensitivity interval. Plotting the new phase after treatment as a function of the old phase (see methods for details) produced PTC s (Johnson, 1992) which further visualized the differences between VEH and NMDA treatments (Figure 1D).

The overall amplitude of the SCN oscillations changed in response to the NMDA application (Figure 1E). The extent of the amplitude changes varied depending on the phase of the clock at the time of the NMDA application, following approximately circadian (cosinor, *P* < 0.0001, *R*^2^ = 0.2436) pattern. In contrast to NMDA treatment, VEH-induced changes in amplitude were randomly distributed along the time axis (cosinor, *P* = 0.85, *R*^2^ = 0.0081). The NMDA-induced pattern likely reflects the phase dispersion among individual SCN cells (An et al., 2013) that increases proportionately with the magnitude of the phase shift, facilitates the entrainment (Abraham et al., 2010) and can lead to almost undetectable overall amplitude as discussed in section NMDAR Activation During a Narrow Window of SCN Sensitivity Can Disrupt Overall PER2 Rhythm. During the dead zone of the PRC, NMDA did not produce significant phase shifts but decreased the phase dispersion between SCN cells, increasing the overall degree of synchronization. Similar reciprocal relationship between phase shift and amplitude change has been previously described in a system of uncoupled oscillators formed by light-sensitive fibroblasts (Pulivarthy et al., 2007; Ukai et al., 2007). In the rat SCN, 12 h long light stimulus can also dramatically decrease the overall amplitude of *Per1* and *Per2* rhythmic expression rhythms *in vivo* (Ukai et al., 2007).

To summarize the results, we have described detailed phase and amplitude effects of NMDAR activation and, for the first time, demonstrated the “singularity” behavior of PER2 luminescence rhythm in cultured SCN slices in response to NMDA, a light-mimicking stimulus.

### Endocannabinoid Signaling Modulates Phase Response to NMDAR Activation in a Time-Specific Manner

Next, in light of the available behavioral (Sanford et al., 2008; Acuna-Goycolea et al., 2010) and electrophysiological (Acuna-Goycolea et al., 2010) evidence, we have used analysis of PER2 rhythm in cultured SCN slices to test whether the EC signaling plays any role in rhythm generation and entrainment.

**FIGURE 2:**
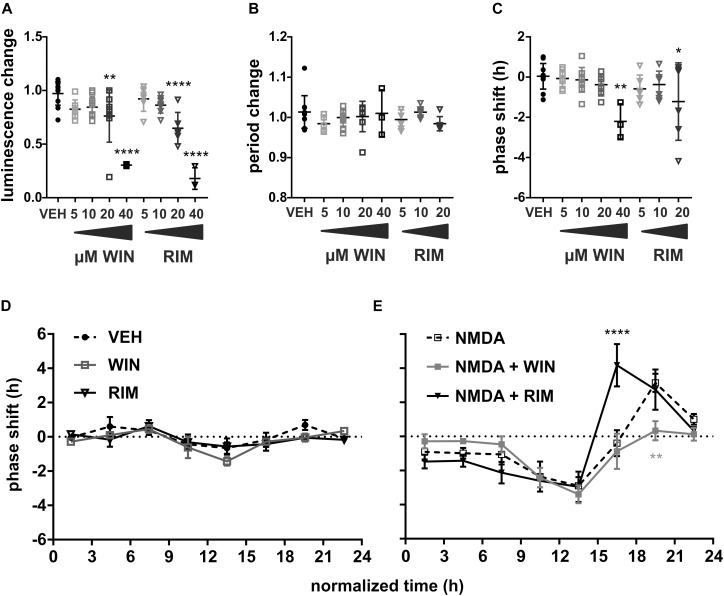
Modulation of SCN phase response to NMDA by endocannabinoid signaling. VEH (0.04% DMSO, *n* = 11, circles), WIN (5–40 μM WIN 55,212-2 mesylate, CB1R agonist, *n* = 3–10/concentration, squares) or RIM (5–40 μM rimonabant hydrochloride, CB1R antagonist, *n* = 6–8/concentration, triangles) were applied to the medium and their effects on PER2 luminescence levels **(A)**, endogenous period **(B)**, and phase **(C)** were analyzed. Asterisks show results of Dunnett’s multiple comparisons test between VEH and the drugs. **(D)** PRCs were constructed as in Figure 1C. VEH (black circles, *n* = 3–8/time point), 10 μM WIN (gray squares, *n* = 3–7/time point, *n* = 2 at *t* = 9–12 h bin) or 10 μM RIM (black triangles, *n* = 3–7/time point, *n* = 2 at *t* = 12–15 h bin) were applied throughout the complete circadian cycle. **(E)** Application of 10 μM WIN (gray squares, *n* = 3–8/time point) or 10 μM RIM (black triangles, *n* = 3–11/time point) preceded the treatment with NMDA by 30 min; NMDA PRC (empty squares) from Figure 1C included for comparison. Asterisks show results of Šidák’s multiple comparisons test between phase shifts in response to NMDA alone vs. WIN/RIM + NMDA at corresponding time points. ^∗^*P* < 0.05, ^∗∗^*P* < 0.005, ^∗∗∗∗^*P* < 0.0001.

First, we have applied a range of concentrations of either CB1R agonist (WIN, time of application *t* = 15–23) or CB1R inhibitor (RIM, *t* = 12–19) on cultured SCN explants and analyzed the effects on PER2 rhythm. The drugs used in final concentration between 5 and 10 μM had no effect on either the luminescence level (Figure 2A), period (Figure 2B), or phase (Figure 2C). At 20 μM, there was a significant decline in luminescence levels for both WIN [Dunnett’s multiple comparisons test, drug vs. vehicle, *q*(53) = 3.48, *P* = 0.0076] and especially for RIM [*q*(53) = 4.90, *P* < 0.0001], which likely influenced the detectable phase delay caused by 20 μM RIM [*q*(50) = 2.94, *P* = 0.0309]. At 40 μM, both WIN [*q*(53) = 7.86, *P* < 0.0001] and RIM [*q*(53) = 9.33, *P* < 0.0001] caused a very large decline in circadian luminescence levels, which also manifested as a significant phase delay for WIN [*q*(50) = 4.08, *P* = 0.0011]. The highest concentration (40 μM) of RIM decreased the luminescence levels by almost 80%, making the analysis of circadian parameters impossible.

We reasoned that the sharp drop of circadian luminescence was likely indicative of a decreased viability of the explants due to high dose of the drugs. To test this further, we used a simple oscillatory model system, which allowed us to analyze the cell viability efficiently and quantitatively. The model was composed of a monoclonal human cell line U-2 OS expressing luciferase under the control of a partial regulatory sequence of clock gene *Bmal1*. WIN (1.25–10 μM) decreased the absolute levels of *Bmal1*-driven luminescence at the highest concentration, echoing its effect on the SCN (Supplementary Figure S1A). The *Bmal1* peak expression was highly correlated (Supplementary Figure S1B, linear regression, *R*^2^ = 0.84, *P* < 0.0001) with the cell viability measured by an ATP assay. Although the models differed in many crucial aspects, such as species, cell type, levels of membrane cannabinoid receptors and the overall sensitivity to the drug, the experiment provided supporting evidence for the close relationship between the clock gene-driven luminescence and the cell viability. Therefore, to avoid any drug toxicity in subsequent experiments, we used both WIN and RIM at the highest concentration that did not significantly decrease the luminescence levels in the SCN.

To verify that we did not miss any possible effect of CB1R activation or inhibition on circadian phase due to a wide time window of drug application, we applied 10 μM WIN, 10 μM RIM or a corresponding vehicle at multiple time points throughout the circadian cycle and constructed a complete PRC (Figure 2D). There was no significant difference between both WIN [2-way ANOVA, *P*_*interaction*_ = 0.2696, *P*_drug_ = 0.0676, *F*(1, 57)_drug_ = 3.47] or RIM [*P*_interaction_ = 0.4685, *P*_drug_ = 0.3401, *F*(1, 53)_drug_ = 0.93] and the VEH. The lack of RIM effect on the phase is not in agreement with results of a previous study, which reported a small phase delay in mouse spontaneous locomotor activity rhythm after *in vivo* infusion with AM 251, another potent CB1R antagonist (Acuna-Goycolea et al., 2010). It is problematic to compare data from *in vivo* and *in vitro* experiments, however, unlike RIM, AM 251 also acts as an agonist of GPR55 receptor (Ryberg et al., 2007). SCN or connected sites expressing GPR55 (Marichal-Cancino et al., 2017) could be involved in the observed behavioral phase shifts. Our data nevertheless suggest that EC signaling via CB1R does not play a direct role in rhythm generation in the SCN.

More interestingly, localization of the CB1R in the vlSCN neurons (Acuna-Goycolea et al., 2010) together with the effects of EC on circadian wheel-running activity in mice (Acuna-Goycolea et al., 2010) and hamsters (Sanford et al., 2008) suggested that EC signaling might play a role in light entrainment. The detailed PRC constructed in the previous experiment allowed us to reveal the effects of activation of CB1R by WIN as well as inhibition of CB1R by RIM on the NMDA-induced phase effects. WIN pretreatment attenuated the phase shifts in response to NMDA, but the effect was restricted to the interval marked by the decline of PER2 levels, resulting in a reduction of phase advances of the SCN rhythm. Construction of a detailed PRC to WIN+NMDA (Figure 2E, gray line) confirmed that compared to NMDA (Figure 2E, dashed line), WIN pretreatment significantly reduced NMDA-induced phase advances during *t* = 18–21 bin [Šidák’s multiple comparisons test, *P*_18–21_ = 0.0027, *t*(107)_18–21_ = 3.79], corresponding roughly to the second half of the subjective night *in vivo* (Field et al., 2000). Therefore, our data are in agreement with the previously published *in vivo* results in hamsters that showed inhibition of phase advance of wheel running activity rhythm after injection of another CB1R agonist, CP55940 (Sanford et al., 2008). Furthermore, our results show that inhibition of CB1R by RIM pretreatment (Figure 2B, black line) caused rather the opposite effect, significantly increasing the magnitude of the phase advance during *t* = 15–18 [*P*_15–18_ < 0.0001, *t*(118)_15–18_ = 5.11] when compared with NMDA alone.

Available electrophysiological data recorded from individual SCN neurons suggest that CB1R activation results in presynaptic suppression of GABA release from axonal terminals (Acuna-Goycolea et al., 2010), whereas glutamate release from RHT remains unaffected. GABAergic signaling in the SCN plays a complex role (Liu and Reppert, 2000). It can be both inhibitory and excitatory (DeWoskin et al., 2015) depending on the balance of chloride influx by Na^+^-K^+^-Cl^−^ cotransporter 1 (NKCC1) and chloride efflux by K^+^-Cl^−^ cotransporters (KCCs), which is determined by their rhythmically regulated expression (Choi et al., 2008; McNeill et al., 2018; Olde Engberink et al., 2018). Our data provide supporting evidence, that circadian phase plays a major role in response to EC and possibly to GABA signaling (Wagner et al., 1997) in the SCN. This could be due to rhythmic expression of CB1R or due to rhythmic regulation of its downstream signaling. Recently, CB1R was suggested to be under circadian regulation in the liver (Bazwinsky-Wutschke et al., 2017), adrenal glands (CircaDB database, Zhang et al., 2014) and possibly in the SCN as well (SCNseq database, Pembroke et al., 2015). Alternatively, we might also attribute it to the effects that both endogenous cannabinoids and related synthetic drugs such as WIN exert on other rhythmically expressed receptors. For example, WIN partially targets nuclear receptors PPARα and γ (Sun et al., 2006; O’Sullivan, 2016), which provide an important metabolic input to the clockwork (Yang et al., 2006; Chen and Yang, 2014). Interestingly, a related isoform PPARβ/δ was shown to be rhythmically expressed in the hamster SCN, where it plays a role in glutamatergic signaling (Challet et al., 2013), though its interaction with WIN or RIM has not been described. Finally, off-target activity of WIN and RIM on unrelated receptors and enzymes (Soethoudt et al., 2017) in the SCN cannot be excluded.

## Conclusion

In conclusion, we have shown here that EC signaling modulates the ability of SCN clock to entrain to the light-mimicking stimulus *in vitro*. Future studies should focus on identifying the downstream circadian component of the EC signaling in the SCN, on monitoring of circadian rhythm with single cell resolution and on *in vivo* experiments to examine further the role of EC signaling in the entrainment of SCN clock. Additionally, it might be interesting to explore potential effects of phytocannabinoids (Whitehurst et al., 2015) and their synthetic analogs on the light entrainment of the human circadian clock, particularly in view of a reportedly common motive for using cannabis as a sleep-facilitating substance (Lee et al., 2007).

## Data Availability

All datasets generated for this study are included in the manuscript and/or the Supplementary Files.

## Author Contributions

MS performed the experiments and wrote the manuscript. AS helped with experiments planning, writing, and reviewing the manuscript.

## Conflict of Interest Statement

The authors declare that the research was conducted in the absence of any commercial or financial relationships that could be construed as a potential conflict of interest.
