# Supplementary material for: Modulation of NMDA-Mediated Clock Resetting in the Suprachiasmatic Nuclei of mPer2Luc Mouse by Endocannabinoids
Source: Front Physiol. 2019 Mar 29;10:361. doi: 10.3389/fphys.2019.00361 (PMC6450388; doi:10.3389/fphys.2019.00361)
Supplement: Supplementary file 1 [file Data_Sheet_1.PDF]

Supplementary Methods

U-2 OS cells (ECACC, Sigma, P<5) were grown in a standard DMEM with 10% fetal calf serum (FCS, Sigma) and 1x GlutaMAX (ThermoFisher) in a 6-well plate, transduced with 0.5ml of ultracentrifuge-concentrated lentiviral particles prepared with a ViraSafe Lentiviral packaging system (Cell Biolabs, USA) according to manufacturer's instructions, which contained pLV6-Bmal-luc circadian reporter (a kind gift from prof. Steven Brown, Zurich, Addgene plasmid # 68833; http://n2t.net/addgene:68833; RRID:Addgene_68833), selected under 10 µg/ml Blasticidin (Sigma) for 5 days, subsequently diluted to 0.5 cell/well to a 96-well plate and clonally expanded under 10 µg/ml Blasticidin for 4 weeks. Monoclonal lines were tested for high luminescence and high rhythm integrity in Lumicycle; monoclonal line no. 5 was used for all subsequent experiments. Cells were grown in a white 384-well plate until confluence, then either 0.1% DMSO, 1.25µM, 2.5µM, 5µM or 10µM WIN 55,212-2 mesylate was applied to the recording medium (luciferin-containing DMEM with higher 10% concentration of FCS but without B27), sealed with a qPCR foil and their luminescence recorded for 10 s every 1h in Luminoskan Ascent (ThermoFisher) plate reader for 6 days. At the end of the experiment, the medium was washed 2x with PBS and ATP levels were analyzed with CellTiter-Glo Luminescent Cell Viability Assay (Promega) according to manufacturer's instructions. Circadian luminescence traces were analyzed by cosinor, mesor of the oscillations during t = 10-34 was expressed as a function of ATP levels and fitted with linear regression using Prism 7 (Graphpad).

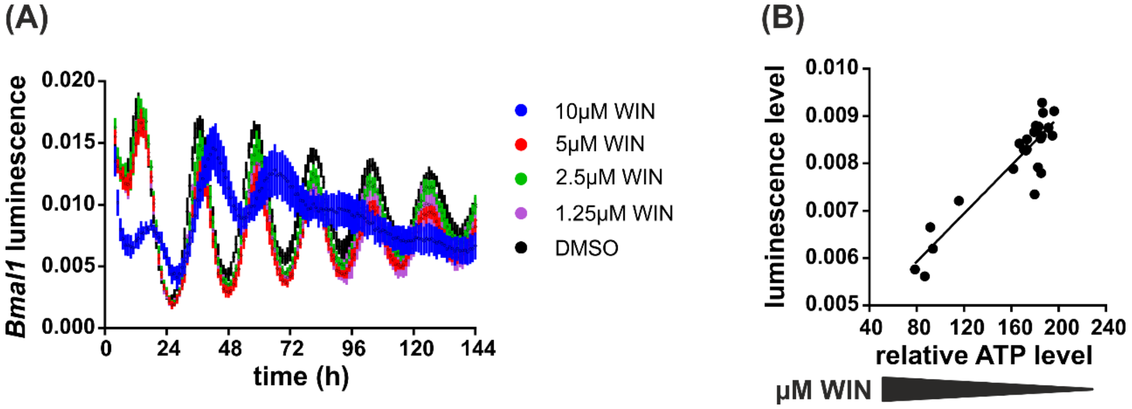

Supplementary Figure S1. (**A**): Circadian rhythm of *Bmal1*-driven reporter in U-2 OS cells is negatively influenced by higher concentrations of WIN (mean ± SD, n = 4-8 wells/ concentration). (**B**): *Bmal1* luminescence level is correlated with cell viability expressed as relative ATP level and affected by increasing levels of WIN.
